# Recombinant insulin-like growth factor-1 activates satellite cells in the mouse urethral rhabdosphincter

**DOI:** 10.1186/1471-2490-13-62

**Published:** 2013-11-26

**Authors:** Wenjie Wei, Pamela S Howard, Edward J Macarak

**Affiliations:** 1 Department of Anatomy and Cell Biology, University of Pennsylvania, 240 S. 40th Street, Philadelphia, PA 19104, USA; 2 Department of Urology, Second Affiliated Hospital of Wenzhou Medical University, 109 W. Xueyuan Road, Wenzhou, Zhejiang 325027, P. R. China

**Keywords:** Recombinant insulin-like growth factor-1, Satellite cells, Activation, Urethral rhabdosphincter, Mouse model

## Abstract

**Background:**

The goal of this study is to demonstrate the efficacy of a new method for the treatment of urinary incontinence by stimulation of urethral rhabdosphincter satellite cells. We show that satellite cells do exist in the sphincter muscle of retired male mice breeders by staining for c-Met, a satellite cell specific protein. Once activated by recombinant mouse Insulin-like Growth Factor-1(rIgf-1), the satellite cells develop into muscle cells within the rhabdosphincter thereby potentially strengthening it.

**Methods:**

20 μl (1 μg/μl) of rIgf-1 was surgically injected directly into the urethral wall of retired male mouse breeders. Mice injected with phosphate buffered saline (PBS) were used as controls. 4 weeks later, urethras were harvested and serially-sectioned through the sphincter for routine hematoxylin-eosin staining as well as immunohistochemical staining with satellite cell specific anti-c-Met antibody and proliferation specific anti-Ki-67 antibody.

**Results:**

Anti-c-Met antibody positive cells (c-Met^+^) were identified in the rhabdosphincter. c-Met^+^ cells increased by 161.8% relative to controls four weeks after rIGF-1 injection. Anti- Ki-67 antibody positive cells were identified and characterized as cells with centrally located nuclei in striated muscle bundles of rIGF-1 treated animals.

**Conclusions:**

Satellite cells in the mouse rhabdosphincter can be activated by rIGF-1 treatment, which subsequently are incorporated into existing skeletal muscle bundles. Using this approach, the rhabdosphincter can be induced to regenerate and potentially strengthen via satellite cell activation and likely improve urinary continence.

## Background

Incontinence affects both adult females and males. In the adult population, urinary incontinence affects an estimated 35% of women 65 years or older and 10% of women younger than 65 years. An estimated 22% of men 65 years or older and 1.5% of men younger than 65 and 30 to 50% of institutionalized adults 65 years or older also have some form of urinary incontinence [1].

Many studies have underscored the role of the rhabdosphincter in mediating continence. Although stress urinary incontinence (SUI) is a common form of incontinence that primarily affects women, its pathophysiology has remained elusive. It is likely, however, that anatomical factors such as urethral hypermobility may contribute to urethral incompetence. Hilton et al. found that the degree of sphincter weakness, as evidenced by decreased maximum urethral closure pressure (MUCP), correlates with the severity of SUI [2]. Rhabdosphincter volume is significantly smaller on ultrasound examination in SUI woman compared to continent controls underscoring the role of muscle mass in maintaining continence [3,4]. Strasser found that the dramatic decrease in the number of striated muscle cells in the rhabdosphincter of elderly patients, owing to apoptosis, represents the morphological basis for high incidence of incontinence in that population [5]. Also, Perucchini et al. found that the number and the density of urethral striated muscle fibers decline with age [6]. Thus, SUI correlates with loss of closing pressure which in turn appears related to striated sphincter muscle mass.

Radical prostatectomy is widely employed as primary treatment for localized prostate cancer. The rates of post-prostatectomy incontinence range from 5%-65% and have significant impact on patient quality of life issues [7-10]. Post- prostatectomy incontinence may be attributed to sphincter dysfunction that occurs as a direct result of surgical injury during the radical prostatectomy [11-13].

Duloxetine, a potent serotonin and norepinephrine re-uptake inhibitor, stimulates the pudendal nerve output to the striated urethral sphincter as a result of the increased levels of serotonin and norepinephrine in the pudendal nerve nucleus in the sacral spinal cord [14]. Clinically, significant improvements in patients suffering from SUI have been demonstrated in studies by Ghoniem et al. [15]. Thus, we can presume, on the basis of these studies, that incontinence can either be improved or cured by enhancing the quantity and/or quality of functioning striated urethral muscle.

Satellite cells, the skeletal muscle stem cells, have been identified in a variety of muscle tissues. It has been demonstrated that skeletal muscle satellite cells, if appropriately stimulated, for example, by IGF-1, will proliferate and differentiate into mature skeletal muscle cells [16-18]. The rejuvenation of satellite cells in the aged rhabdosphincter has been previously demonstrated [19,20]. In this study, we show that satellite cells do exist in the sphincter muscle of retired male mice breeders by staining for c-Met, a satellite cell specific protein which is the receptor for Hepatocyte Growth Factor (HGF) [17,21]. Once activated, the satellite cells differentiate into muscle cells within the rhabdosphincter.

## Methods

### Animal model

Retired (4 month old) Swiss Webster mice (Charles River), weighing 30–46 grams, were used. A low abdominal midline incision was made under isoflurane anesthesia.10 μl of 1 μg/μl recombinant mouse IGF-1 (R&D system, 791-MG) was injected, using a Hamilton micro-injector with 30G needle, in the each lateral side of the urethra after its surgical exposure. After injection of IGF-1, the incision was closed, followed by administration of 0.1 ml 0.03 mg/ml buprenophine to relieve post-surgical pain. 4 weeks after surgery, the mice were sacrificed and the urethral tissue was harvested for analysis. Sham-operated controls were treated identically except that PBS was injected in place of IGF-1. Three mice were used in each group. The animal study was approved by IACUC of University of Pennsylvania.

### Histological analysis

The lower urinary tract from the bladder to the glans penis was carefully dissected and removed for histological analysis. Initially, to study potential rIGF-1 accessibility to satellite cells in the muscle bundle, Indian ink was locally injected into the urethral wall in the region just below the prostate. The urethra was serially sectioned at 7um for routine H& E staining. Muscle bundles containing central nuclei were assumed to be new muscle bundles.

### Immunohistochemistry

Urethras were excised and embedded in O.C.T for cryo-sectioning. To detect c-Met, the sections were swollen by treatment with 0.5 M acetic acid for 6 hours to expose c-Met antigen and rinsed with PBS. Sections were then incubated with goat anti-mouse c-Met antibody in a humidified chamber (R&D. AF527) 1:100 at 4°C overnight, followed by 3 PBS washes and incubation with Alex fluor-594 linked rabbit anti-goat IgG-fab 1: 200 dilution (Invitrogen) for 1 hour. To detect ki-67, sections were incubated with 1:600 dilution rabbit anti-human Ki67 (NCL-ki67p polyclonal, Novo Castro) at 4°C overnight. Sections were then washed 3× with PBS and incubated with Alex fluor-594 linked goat anti rabbit IgG-fab, 1: 200 dilution, (Invitrogen) for 1 hour at room temperature. The sensibility and specificity of the antibodies used in these studies have been confirmed by staining mouse leg skeletal muscle tissue. Nuclei were stained by Chromomycin A_3_ (0.1 mg/ml, Sigma) for 10 minutes at room temperature. Using fluorescence microscopy, cells found at the periphery of muscle bundles stain red with c-Met antibody and Alex Flour-594 conjugated secondary antibody demonstrating that they are satellite cells. Nuclei were stained with chromomycin A_3_, which appears green. The co-localization of c-Met and chromomycin appears yellow. Yellow cells were thus identified as satellite cells. To quantify the number of satellite cells in the U-RS, c-Met positive cells were counted in 3 random fields from each section (3 sections per animal). Images were captured using a Nikon Digital Camera system. Image J was software was used to align the individual images to demonstrate the co-localization of either c-Met or Ki67 positive staining with nuclei.

### Statistical analysis

*one-ANOVA and student-t test* were used to determine significance among and between 2 groups, respectively.

## Results

### Anatomy and surgical exposure

**Figure 1:**
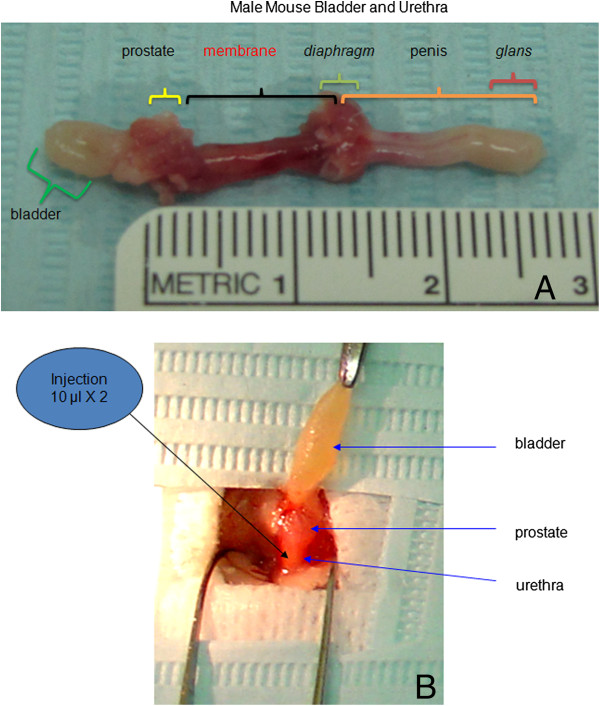
**Anatomy and surgical exposure. A**: Male mouse urethra comprises 3 parts, an anatomy which is similar to that found in humans: the prostatic urethra, the membrane urethra and the spongy urethra. The membrane urethra is the section between the prostate and diagram, it is approximately 0.8 to 1.2 cm in length and it can be easily surgically exposed for local injections. **B**: After a low abdominal mid-line incision was made, the male mouse urethra was exposed for injection in the lateral aspects of the urethra.

The 42 g Swiss Webster male mouse urethra is about 2.7 cm in length and consists of 3 parts which are similar to those found in humans: the prostatic urethra, the membrane urethra and the spongy urethra. The membrane urethra is the section below the prostate and above the diagram and is approximately 0.8 to 1.2 cm long and can be surgically exposed for local injection (Figure 1A, 1B).

### Locally injected rIGF-1 accessibility to satellite cells

**Figure 2:**
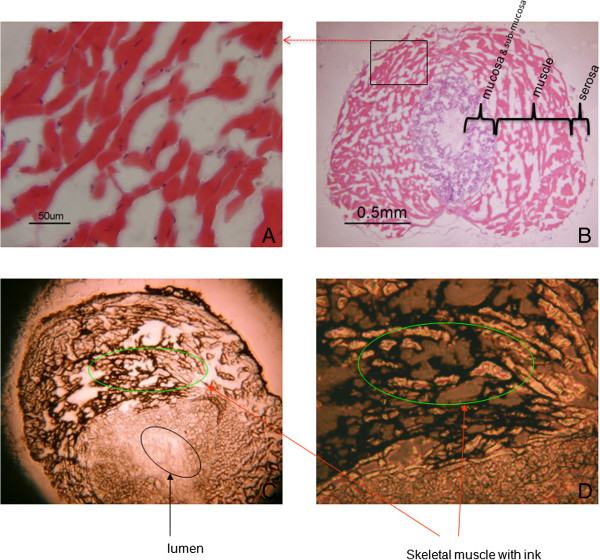
**Locally injected rIGF-1 accessibility to satellite cells in male mouse urethra rhabdosphincter. A**, **B**: Cross section of the male membrane urethra. The inner layer consists of mucosa and sub-mucosa, the outer layer is a serosa and the middle layer is muscle layer contains the rhabdosphincter muscle goes along and surrounds the urethral lumen. **C**, **D**: Injection of India ink into the urethral rhabdosphincter. The rhabdosphincter was infiltrated by locally injected India ink, demonstrating potential accessibility of injections to satellite cells within the muscle.

For histological analysis, the urethra was serially sectioned at 7 um for normal H & E staining. Three layers which surround the urethral lumen can be demonstrated. The inner layer is combined mucosa and sub-mucosa, the outer layer is serosa. The middle layer is the urethral rhabdosphincter (U-RS) muscle layer, it contains the rhabdo-muscle which extends longitudinally and surrounds the urethral lumen. (Figure 2A, 2B) To demonstrate potential rIGF-1 accessibility to satellite cells in the U-RS, India ink was locally injected into the wall of membrane urethra (Figure 2C, 2D).

### Satellite cells (c-Met^+^) exist in retired male mouse U-RS

**Figure 3:**
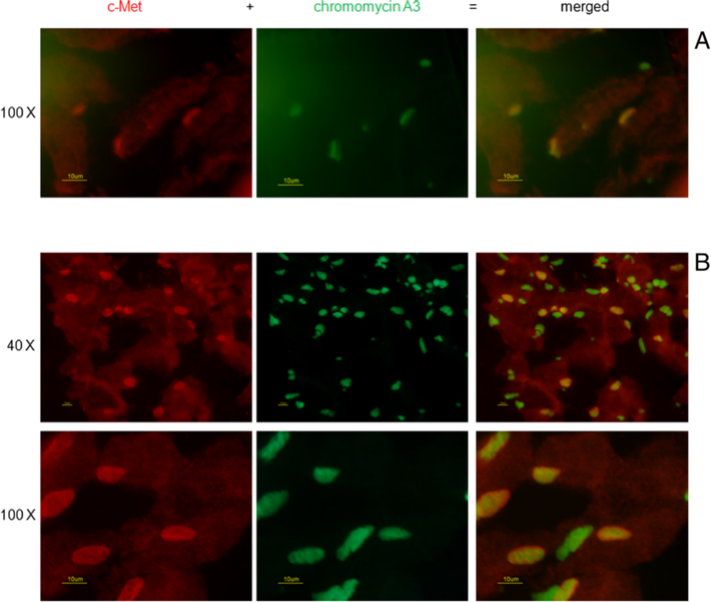
**Satellite cells (c-Met**^**+**^**) exist in retired male mouse urethral rhabdosphincter.** There is positive staining with anti-c-Met antibody at the muscle periphery bundle demonstrating the presence of satellite cells (red). Nuclei were stained with chromomycin A_3_ (green). Merged images show co-localization (yellow). **A** non-operated control; **B**: rIGF-1 Injected urethral rhabdosphincter.

To detect and demonstrate the presence of satellite cells, the U-RS was stained with an antibody to c-met. All nuclei, including those of satellite cells were stained with Chromomycin A_3._ Satellite cells, however, were thus double stained with both Chromomycin A and c-met while non satellite cell only stained with Chromomycin A as shown in Figure 3.

### Stimulation of satellite cells (c-Met^+^) after rIGF-1 treatment

**Figure 4:**
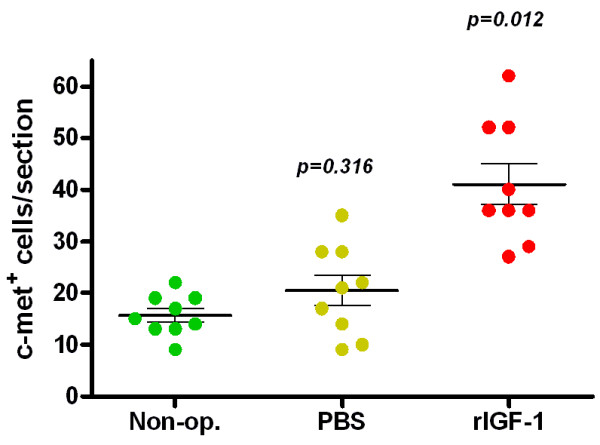
**Anti-c-Met positive satellite cells in animals treated with rIGF-1.** In urethras injected with rIGF-1, the number of c-Met + satellite cells increased significantly versus PBS-injected or non-operated controls. Four weeks after treatment with rIGF-1, c-Met^+^ satellite cells increased by 161.8% (41.1 ± 5.4 verse 15.7 ± 1.3, p = 0.012), while the sham operated (PBS) group showed no significant change (20.4 ± 3.9 verse 15.7 ± 1.3, p = 0.316).

Mice U-RS were injected with rIGF-1 and sacrificed after 4 weeks of treatment. To quantify the number of satellite cells in the U- RS, c-Met positive cells were counted in 3 random fields from each section (3 sections per animal). The numbers of c-Met positive cells in the U-RS of IGF-1 treated, sham-operated and non-operated animals are shown in Figure 4. Four weeks after treatment with rIGF-1, satellite cells (c-met^+^) increased by 161.8% (41.1 ± 5.4 verse 15.7 ± 1.3, p = 0.012), comparing to the non-operated controls; while the sham-operated (PBS) group showed no significant change from non-operated controls (20.4 ± 3.9 verse 15.7 ± 1.3, p = 0.316).

### Proliferation of satellite cells were demonstrated Ki-67^+^ after rIGF-1

**Figure 5:**
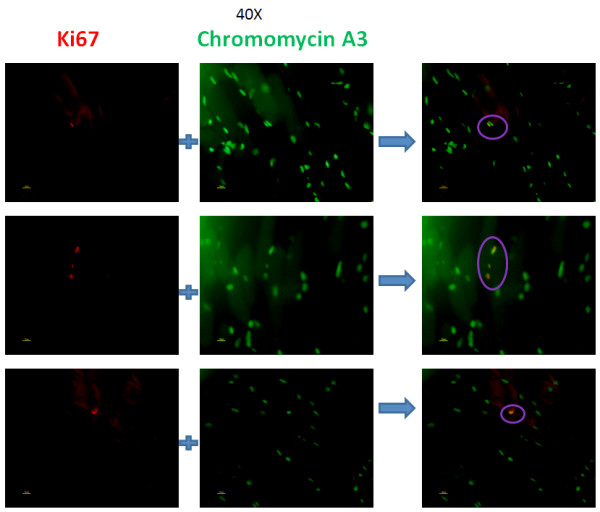
**Proliferation of satellite cells after rIGF-1 injection.** Cells within the rhabdosphincter stained with anti-ki67 antibody (red) indicating cell proliferation. Nuclei were stained with chromomycin A_3_ (green). Note the co-localization (yellow) in the merged photograph.

To demonstrate that the increase in the number of the satellite cells in the U-RS was the result of de-novo mitosis after stimulation by rIGF-1, ki-67 antibody staining was carried out. Ki-67 is a protein which is only expressed in dividing cells [22] and staining with an antibody to Ki-67 was used to detect the mitotic activity arising after injection of the U-RS with rIGF-1. To obtain the total number of cells within the U-RS, all nuclei were stained with chromomycin A_3_. Ki-67 positive cells were found on the peripheral zone of the muscle bundles located with the U-RS as shown in Figure 5.

### New muscle cells were demonstrated with central nuclei in muscle after rIGF-1

**Figure 6:**
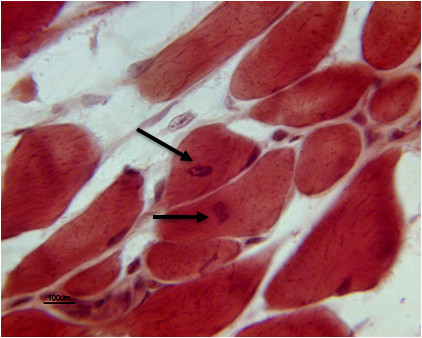
**New muscle cells were incorporated into muscle bundles after rIGF-1 injection.** Skeletal muscle cells with central nuclei are present in the rhabdosphincter muscle bundles (arrows) which have differentiated from satellite cells. Cells with central nuclei were not detected in the controls.

To demonstrate that the satellite cells which arose after rIGF-1 injection differentiated into new striated muscle cells with the U-RS, serial sections of the injected muscle tissue were obtained and stained with H&E. These sections show the presence of skeletal muscle cells with central nuclei, indicating that they represented newly differentiated muscle [23] (Figure 6).

## Discussion

Skeletal muscle is normally a post-mitotic tissue; however, when appropriately stimulated, it can increase in mass. When this occurs, it is predominantly owing to the activation, proliferation and fusion of satellite cells. Satellite cells, which are muscle-derived stem cells, are believed to be pluripotent, and when appropriately stimulated, divide and become assimilated within exiting myofibers [16,17,24-26]. They are normally quiescent and reside beneath the basal lamina of adult skeletal muscle closely juxtaposed against mature skeletal muscle fibers. The satellite cell population accounts for 2-5% of total nuclei in adult muscle [27,28]. As demonstrated in these studies, they can be activated by treatment with rIGF-1 to differentiate into muscle tissue to, in effect, regenerate and increase the mass of muscle [27]. Since the U-RS is believed to weaken in aging adults, and since a weakened U-RS is believed to underlie many of the symptoms associated with stress urinary incontinence, treatment of affected individuals with rIGF-1 has great potential for improving their quality of life by strengthening the rhabdosphincter.

The human rhabdosphincter (RS) is a collar of skeletal muscle which surrounds the urethra, permitting it to clamp shut closing off the flow of urine from the bladder. The damage to the RS during prostatectomy can result in male incontinence. Similar to the human, the male mouse urethra contains a segment between the prostate and the urogenital diagram which is surrounded by sphincteric muscle whose contraction leads to the closure of urethra. This segment can be easily surgically exposed for local injection in the mouse.

In these studies, we have demonstrated the existence of satellite cells in the mouse U-RS which can be induced to divide and differentiate into muscle tissue by injection of rIGF-1. We chose retired, older male mice for these experiments to demonstrate that the satellite cells being activated were not merely growing as a result of developmental activities. However, we have also carried out similar experiments with younger mice with similar results (data not shown). The importance of growth factors, such as IGF-1, in promoting skeletal muscle hyperplasia has been previously reported [29,30]. These results show that the mouse model can be utilized to further investigate the efficacy of this treatment for other types of incontinence since the satellite cell response was robust (approximately 161.8% increase in the number of satellite cells).

New muscle fibers, characterized by central nuclei, appeared 4 weeks after rIGF-1 treatment further validating the concept that the activated satellite cells become incorporated into existing muscle, increasing muscle mass. The repair and/or regeneration of new muscle within damaged or aged U-RS to improve urinary incontinence.

Because this study was carried out using an animal model with a small sample size, further functional studies of changes in muscle contractility/physiology and urodynamic changes which occur after treatment will need to be carried out to further validate these data. Nevertheless, the basic concept that treatment of the U-RS with rIGF-1 can increase its muscle mass has been demonstrated, indicating that this approach is potentially useful since current treatments for stress urinary incontinence (sling, suspension, and collagen bulking) are neither satisfactory nor long-lasting.

## Conclusion

Satellite cells (muscle stem cells) in the aged murine rhabdosphincter can be activated with rIGF-1 which then develop into new muscle cells and which become incorporated into pre-existing muscle tissue. This animal model can be used to develop new methods to regenerate and strengthen the urethra and potentially alleviate the incontinence associated with many uropathies whose root cause is a weakened rhabdosphincter.

## Abbreviations

U-RS: Urethral rhabdosphincter; rIGF-1: Recombinant insulin-like growth factor −1; H&E: Hematoxylin and eosin; PBS: Phosphate buffered saline.

## Competing interests

The authors declare that they have no competing interests.

## Authors’ contributions

WW carried out all studies and drafted the manuscript. PSH and EJM made substantial contributions to conception, data analysis and interpretation, and manuscript revising. All authors read and approved the final manuscript.

## Pre-publication history

The pre-publication history for this paper can be accessed here:

http://www.biomedcentral.com/1471-2490/13/62/prepub
